# Orthopaedic and trauma surgeons’ prioritisation of app quality principles based on their demographic background

**DOI:** 10.1186/s12891-023-06226-y

**Published:** 2023-02-23

**Authors:** Christin Malinka, Florian Dittrich, David Alexander Back, Jörg Ansorg, Ute von Jan, Urs-Vito Albrecht

**Affiliations:** 1 Peter L. Reichertz Institute for Medical Informatics of TU Braunschweig and Hannover Medical School, Hannover Medical School, Hannover, Germany; 2 Joint Centre Bergischland, Sana Fabricius Clinic Remscheid, Remscheid, Germany; 3 Department of Digital Medicine, Medical Faculty OWL, Bielefeld University, Bielefeld, Germany; 4 Department for Traumatology and Orthopedics, Bundeswehr Hospital Berlin, Berlin, Germany; 5 Center for Musculoskeletal Surgery, Charité – Universitätsmedizin Berlin, Berlin, Germany; 6 Professional Association of Specialists in Orthopaedic and Trauma Surgery, Berlin, Germany

**Keywords:** Mobile health apps, Quality principles, Kano survey, Prioritisation, Orthopaedics and trauma surgery, Physicians

## Abstract

**Background:**

Although apps are becoming increasingly relevant in healthcare, there is limited knowledge about how healthcare professionals perceive “quality” in this context and how quality principles that can aid them in assessing health-related apps may be prioritised.

The objective was to investigate physicians’ views of predefined (general) quality principles for health apps and to determine whether a ranking algorithm applied to the acquired data can provide stable results against various demographic influences and may thus be appropriate for prioritisation.

**Methods:**

Participants of an online survey of members of two German professional orthopaedics associations conducted between 02/12/2019 and 02/01/2020 were asked about their perception of a set of quality principles for health apps (i.e., “practicality,” “risk adequacy,” “ethical soundness,” “legal conformity,” “content validity,” “technical adequacy,” “usability,” “resource efficiency,” and “transparency”). Structured as a Kano survey, for each principle, there were questions about its perceived relevance and opinions regarding the presence or absence of corresponding characteristics. The available data were evaluated descriptively, and a newly developed method for prioritisation of the principles was applied overall and to different demographic strata (for validation).

**Results:**

Three hundred eighty-two datasets from 9503 participants were evaluated. Legal conformity, content validity, and risk adequacy filled ranks one to three, followed by practicability, ethical soundness, and usability (ranks 4 to 6). Technical adequacy, transparency, and resource efficiency ranked last (ranks 7 to 9). The ranking based on the proposed method was relatively stable, irrespective of demographic factors. The principles were seen as essential, with one exception (“resource efficiency”). Only those with little to no interest in digitisation (22/382, 5.8%) rated the nine principles indifferently.

**Conclusions:**

The specified quality principles and their prioritisation can lay a foundation for future assessments of apps in the medical field. Professional societies build upon this to highlight opportunities for digital transformations in medicine and encourage their members to participate.

**Supplementary Information:**

The online version contains supplementary material available at 10.1186/s12891-023-06226-y.

## Background

The German Digital Healthcare Act [1], passed in December 2019, introduced various measures to promote the digitisation of healthcare. Among other things, this act made it possible to prescribe (and obtain reimbursement for) health apps for those enrolled in statutory health insurance in Germany. Only apps rated as low-risk medical devices (i.e., class I and IIa [2]) that additionally must be listed in a directory of eligible apps provided by the German Federal Institute for Drugs and Medical Devices (BfArM) may be prescribed [1, 3]. To be approved, apps must conform to established data protection and security standards and demonstrate evidence of medical benefit. As of May 2022, there were only 33 apps listed in the BfArM’s directory [4]. Still, apps and smartphones are not yet integral to healthcare processes in Germany. This is caused by several uncertainties among healthcare professionals, manufacturers, and patients due to a lack of evidence on health apps’ efficacy and economic aspects [5, 6]. The assessment of the quality of health apps and their suitability for individual patients is complex [7–9]: the concept of quality is not widely consensualised [10], and, moreover, manufacturers rarely provide transparent enough information about quality-relevant aspects [11, 12]. Tools that aim to facilitate quality assessments may be perceived as inappropriate, too complex, or too burdensome; thus, no approach has been successfully established [7]. Therefore, creating appropriate support tools based on meaningful quality principles for the target group seems imperative. We aimed to develop a method that may allow paring down such lists through prioritisation, ideally explicitly adapted to the respective target group.

Therefore, we present an exemplary, more detailed evaluation of the previously introduced prioritisation method for quality criteria based on a Kano survey [13]. Specifically, we evaluated the feasibility of determining an adequate and stable ranking for nine (app) quality criteria for use in health contexts concerning potential demographic influences. The quality criteria had been previously developed [14] and evaluated in two surveys among medical students [12] and members of the German Society of Internal Medicine (DGIM) e.V [11]. In both studies, the participants rated all nine quality principles similarly significant. The app description texts used to assess the compliance of the available information with these quality principles were primarily considered as insufficiently informative, even though the definitions of the quality principles were broad. In addition, participants noted the need to consider app quality in general but that it would be too time-consuming to address all nine quality principles. Therefore, it was desired to reduce the list of quality criteria or to enable a ranking of these to prioritise the most important ones. To this end, we turned to another group of stakeholders and assessed the data obtained using the prioritisation mentioned above [13].

The work required for this is based on an original two-part survey developed by the author team and pretested by several physicians. Part 1, already published in [3], showed that most participants were unfamiliar with the requirements of the Digital Healthcare Act [1]. Also, there was a strong scepticism about prescribing health apps, possibly stemming from fear about their potential (technical and health-related) risks [3]. The second part of the questionnaire, for which detailed results are presented here, dealt with the aforementioned assessment and prioritisation of quality principles for (mobile) health apps.

## Methods

### Study design

Members of the German Society for Orthopaedics and Trauma Surgery (DGOU) e.V. and the Professional Association for Orthopaedics and Trauma Surgery (BVOU) e.V. were invited to an anonymous two-part online questionnaire via the official email distribution lists of these associations. The survey took place online between 02/12/2019 and 02/01/2020 using SoSci Survey on a local installation at Hannover Medical School (version 3.2.00, SoSci Survey GmbH, Munich, Germany). A reminder email was sent after 2 weeks.

### The questionnaire

#### Demographics

The demographic items were age, gender, qualification, professional experience, institution, and state of professional practice (six closed multiple-choice questions). The participants were then asked whether health apps were used for private or work purposes, whether patients had already approached them about health apps, and whether they were interested in digitisation topics (one closed multiple-choice question, three dichotomous yes-no questions, and one free-text question for comments).

#### Selection of the quality principles

The set of quality principles used in this study – namely, “practicality,” “risk adequacy,” “ethical soundness,” “legal conformity,” “content validity,” “technical adequacy,” “usability,” “resource efficiency,” and “transparency” [14] – was curated from existing literature as well as initiatives active in the app quality context and applicable general and health-related software quality standards (e.g., [15–25]). The selected nine quality principles were also pre-evaluated [10–12] in a multi-step process before the commencement of the work presented here. The initial selection process was done in close collaboration with professional medical societies [10] and the Swiss competence and coordination centre for eHealth [14, 26].

#### Survey questions related to the quality principles

We asked the participants to state their perceived relevance of the quality principles (on a 5-point Likert scale ranging from “unimportant” to “very important”) and to additionally rate them using questions based on the Kano model [27–29]. This model is popular in the context of marketing research [30, 31] for assessing customer satisfaction with products. For this purpose, the model evaluates the relationships between specific characteristics implemented in a product (or not). Applying Kano’s method makes it easier to see which product features (potential) customers expect or are more neutral about, which might be rated negatively or trigger an enthusiastic, positive reaction. According to Kano, there is not necessarily a linear relationship between whether a particular feature elicits a positive or negative response and whether it fulfils an actual “need” or not [30, 32]. Kano’s model uses so-called functional and dysfunctional questions to assess satisfaction and dissatisfaction with the features under consideration. These follow patterns such as “What would you think if [ …] were [implemented / available] in the product” (for functional questions), and “What would you think if [ …] were not [implemented / available] in the product” (for dysfunctional questions). Answer options for both types of questions were “I would be very pleased”, “I’d expect this”, “I don’t care”, “I could accept that”, and “That would really bother me”. The complete list of the relevance related as well as the functional and dysfunctional questions we used for assessing the nine quality principles can be found in [13] and Supplementary Tables S1 and S2 (translated from the original German language versions).

To avoid bias due to a specific order of the quality principles, the question blocks with the three questions per quality principle (functional, dysfunctional, relevance related) were randomised for the participants.

### Evaluation

Only completed questionnaires were analysed, and there was a descriptive evaluation of the demographics. Subsequently, according to Kano’s satisfaction model [28, 29], the analysis was carried out per participant and quality principle. According to Kano, a product feature can be rated as either attractive (A), must-be (M), one-dimensional (O), indifferent (I), reverse (R), or questionable (Q). To reflect an individual’s appraisal of the respective feature, the answer combinations for the functional and dysfunctional questions are used (see [13] or [28] for a more detailed explanation as well as a tabulation of the possible answer combinations and their assigned categories). An “attractive” feature (A) increases satisfaction [28]. “Must-be” features (M) are deemed essential or “taken for granted.” They lead to satisfaction if present but cause extreme dissatisfaction if absent [28]. “One-dimensional” (O) represents features that increase satisfaction when fulfilled but dissatisfaction when this is not (entirely) the case [28]. An “indifferent” rating (I) indicates features that increase neither satisfaction nor dissatisfaction if they are available (or not) [33]. Features falling in the “reverse” (R) category negatively impact satisfaction if provided and show increased satisfaction if absent. This may, for example, relate to features that the target group perceives as too complex to be worth it. The last category, “questionable” (Q), refers to features where the answers to the functional as well as dysfunctional question are in apparent contradiction (e.g., if both answers were given as “That would really bother me” [28]). Both the “reverse” as well as the “questionable” category may either indicate a problem with the questions employed (e.g., vague wording) or may even represent a participant’s unwillingness to provide a meaningful answer (for whatever reason). There are various strategies for evaluating the categorisations identified at the individual level to elicit a collective assessment of product features for a more extensive number of participants.

The simplest method applies the category most often assigned to the respective feature within the overall group of participants, which, in our case, except for “resource efficiency”, led to “must-be” ratings [13] and, thus, did not support prioritisation of the principles. Similarly, relying on the per-category counts for all participants would have been problematic. For example, quality principles with a ratio of 51:49 of “attractive” vs “indifferent” ratings would have been rated just as “attractive” as those with fewer “indifferent” ratings. Based on such considerations, in [28], Mike Timko proposed using satisfaction and dissatisfaction coefficients built upon Kano’s work. These “Better” and “Worse” coefficients are calculated as

$$Better=\frac{A+O}{A+O+M+I},{\text{with}}\;0\leq Better\leq1$$

and

$$Worse=-\frac{O+M}{A+O+M+I},{\textrm{with}}-1\le Worse\le 0$$

and describe the relative value of meeting a customer requirement or failing to do so [13, 28]. The Worse-Better pairings thus obtained can be plotted in a more easily interpretable graph, in which the four quadrants represent specific categorisations such as “attractive,” “one-dimensional,” “indifferent,” and “must-be,” while additionally allowing for a better distinction between features depending on their relative positions within the coordinate system. As stated by Timko, when trying to determine which features to keep (or omit), those with higher scores for “Better” should be preferred, as should those with smaller “Worse” values, as these lead to less discontent [13, 28]. Often, the “Worse” and “Better” values are multiplied by the average relevance values (rescaled to 0 to 1) to allow even better discrimination between features that would otherwise be located in direct vicinity within this coordinated system. In the following sections, this is denoted by a subscripted “I,” i.e., “Better_I_” and “Worse_I_.” While using Timko’s approach already facilitated the visual interpretation of the ratings, we were in search of a method allowing a more granular, number-based assessment for ranking and prioritising the quality principles, even in cases where all quality principles are rated similarly based on their assigned category.

For this purpose, the so-called in-line-of-sight approach was developed based on the two coefficients mentioned above [13] and applied to our data. Essentially, this method uses two factors to determine the ranking for a given number of features: On the one hand, it relies on the proximity of the quality principle’s coordinates to the outermost corner of the quadrant or category under consideration, as this corner corresponds to the point most clearly representing the quadrant. On the other hand, the method takes the ratio between the Better and Worse coefficients into account to give preference to features with larger Better coefficients in cases where the distance to the outer corner would otherwise again have led to two features being assigned the same rank [13]. provides a more detailed explanation for the approach.

The in-line-of-sight approach was first applied for the overall group and subsequently for various demographic strata to determine whether demographic aspects possibly impact the ranking of the principles. All calculations and the compilation of the graphics presented here were carried out with R [34] (initially, version 3.6, later on, version 4.1.2), using additional packages (e.g. [35–37]).

## Results

### Demographics

Of the 9503 members of the DGOU and the BVOU we contacted, 382 (4%) completed questionnaires for this part of the survey and were thus included in the analysis. Their demographics are described in Table 1.

**Table 1:** Base demographics for the *N* = 382 participants with fully completed questionnaires in the part of the survey presented here (adapted from [13])

Characteristic	*N* = 382
Age in years, n (%)	
up to 40 years	92 (24.1%)
41 to 60 years	211 (55.2%)
Older than 60 years	79 (20.7%)
Gender, n (%)	
Female	54 (14.1%)
Male	328 (85.9%)
Work experience, n (%)	
Up to 20 years	169 (44.2%)
21 years and longer	194 (50.8%)
Retired	19 (4.97%)
Work setting, n (%)	
Acute care / hospital	232 (60.7%)
Rehabilitation centre	15 (3.93%)
Medical care centre, private practice, other	134 (35.1%)
Not answered	1 (0.26%)
Geographic location, n (%)	
Germany	370 (96.9%)
Other countries	10 (2.62%)
Not answered	2 (0.52%)
Interest in digitisation, n (%)	
Highly interested and interested (aggregated)	316 (82.7%)
Neutral	44 (11.5%)
Little to no interest (aggregated)	22 (5.76%)
Uses health-related apps in private settings, n (%)	139 (36.4%)
Uses health-related apps at work, n (%)	136 (35.6%)
Been asked about an app/recommendation, n (%)	86 (22.5%)

### Analysis

#### Participants overall

**Fig. 1:**
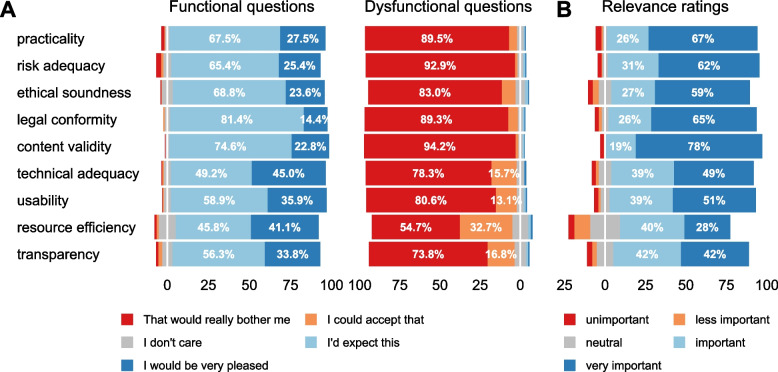
Distribution of the answers given for the functional and dysfunctional questions (**A**) as well as for the perceived relevance (**B**) for each quality principle (all *N* = 382 participants)

Predominantly, the participants rated the nine quality principles as either “important” or “very important” in terms of their relevance (on average, 87.9%, Fig. 1, right). Resource efficiency was rated as least relevant, with only 68% of the participants seeing this quality principle as “important” or “very important” (Fig. 1, right). This is in line with our previous studies in the app quality context, where students of medicine [12] and physicians practising internal medicine [11] assessed the quality principles similarly.

“Legal conformity” (81%) and “content validity” (75%) were considered prerequisites (“I’d expect this”) by more participants than any other quality principle (Fig. 1, left). In contrast, for “resource efficiency” and “technical adequacy” (and, to a somewhat lesser degree, for “usability” and “transparency”), the proportion of those who had expressed that they would expect sufficient consideration in an app was much lower (“resource efficiency,” 45.8%; “technical adequacy,” 49.2%; “usability,” 58.9%; “transparency,” 56.3%), and the proportion of those who rated the respective quality principle being covered as very positive increased (Fig. 1, left).

Insufficient coverage of the quality principles was predominantly (81.8% on average) rated as bothersome (see dysfunctional questions, Fig. 1, centre). The quality principles “content validity” (94.2%), “risk adequacy” (92.9%), “practicality” (89.5%), and “legal conformity” (89.3%) were seen as something to be particularly missed if absent.

**Fig. 2:**
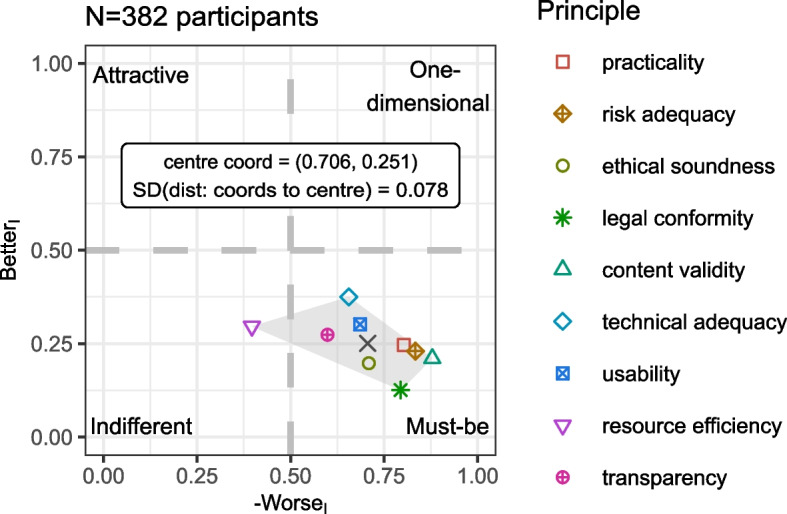
Plot of the satisfaction and dissatisfaction coefficients (Better_I_, Worse_I_) for all nine quality principles (without demographic stratification)

**Fig. 3:**
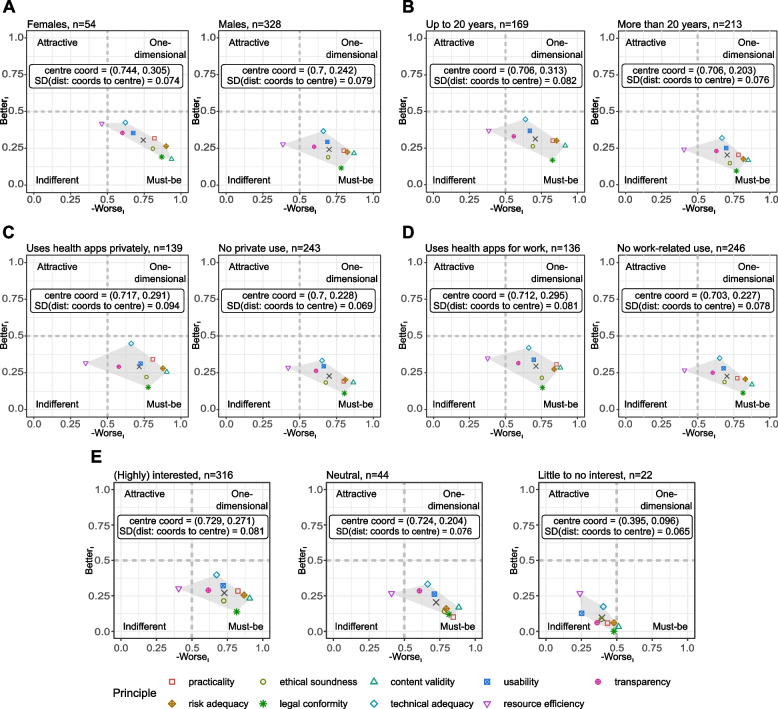
Plots of the Better_I_ and Worse_I_ value pairings for the nine quality principles, for the strata **A** gender (female, male, adapted from [13]), **B** work experience (≤ 20 years, > 20 years incl. retirees), **C** private usage of health apps (yes, no), **D** work-related usage of apps (yes, no) and **E** interest in digitisation (highly interested and interested, neutral, little to no interest)

After calculating the satisfaction coefficients according to Timko [28], taking into account the relevance ratings (as shown in Fig. 1, right), only “resource efficiency” was categorised as indifferent. The other eight principles were rated as “must-be” (Fig. 2, plotted with the values provided in Supplementary Table S3, part A). “Resource efficiency” thus contributed neither to satisfaction nor dissatisfaction. The final differentiation step using the “in-line-of-sight” method, as described in [13], resulted in the following ranking: the participants ranked “legal conformity” (1), “content validity” (2) and “risk adequacy” (3) on the first three places, followed by “practicability” (4), “ethical soundness” (5), “usability” (6) and “transparency” (7), “technical adequacy” (8) and “resource efficiency” (9) on the last three places (Table 2, see in detail Supplementary Table S3, part A for all respondents). Regardless, the centre of gravity across all nine quality principles was near the centre of the “must-be” sector (− 0.706, 0.251) with a standard deviation of the distance between the point coordinates and the centre of gravity of 0.078 (Fig. 2). In the following paragraphs, stratifications for several variables are described. For illustration, Fig. 3, parts A to F, show the plots for the different strata, while the corresponding rankings are listed in Table 2 for reference.

**Table 2:** Ranks for the quality principles, as determined by the “in-line-of-sight” method. A for the overall group of participants, as well as stratified by B gender, C professional experience, D private and E work-related use of health-related apps, and F interest in digitisation. See Supplemental Table S3 for the values used for calculating the respective ranks

Principle	Rank based on											
	A: Participants overall	B: Gender		C: Professional experience in years		D: Private use		E: Work-related use		F: Interest in digitisation		
	–	f	m	≤ 20	> 20	yes	no	yes	no	high	neutral	low
Legal conformity	1	2	1	1	2	1	1	1	1	1	2	2
Content validity	2	1	2	2	1	2	2	3	2	2	3	1
Risk adequacy	3	3	3	3	3	4	4	4	3	3	5	3
Practicality	4	5	4	4	4	5	3	5	4	5	1	4
Ethical soundness	5	4	5	5	5	3	5	2	5	4	4	5
Usability	6	6	6	6	6	6	6	6	6	6	6	8
Transparency	7	7	7	7	7	7	7	7	7	7	8	7
Technical adequacy	8	8	8	8	8	8	8	8	8	8	7	6
Resource efficiency	9	9	9	9	9	9	9	9	9	9	9	9

#### Stratification by gender

Stratified by gender (Fig. 3A), the Worse_I_ and Better_I_ coordinates for most quality principles differed significantly (Euclidean distance between the Worse_I_ and Better_I_ coordinate pairs almost universally greater than 5% or 0.05 ·  √ 2 ≈ 0.0707 of the maximum possible distance represented by the diagonal in the coordinate system), except for technical adequacy and usability. However, there were only minor changes in the rankings obtained for female and male participants compared with the overall group (Table 2 and Supplemental Table S3). Among female participants (*n* = 54), “content validity” was in the first place, and “legal conformity” was in second place; among male respondents (*n* = 328), the order was reversed. The same was true for “ethical soundness” and “practicality” (ranks 4 and 5, respectively). Like the non-stratified results, apart from “resource efficiency,” all quality principles were again found in the “must-be” quadrant. While placed in the “indifferent” quadrant for both groups, “resource efficiency” was located closer to the neutral area (i.e., absolute values for Better_I_ and Worse_I_ closer to 0.5) for the female participants. Likewise, the perceptions of the principles were shifted more toward the “one-dimensional” quadrant for them compared to the male participants (see Fig. 3A, plotted with values from Supplementary Table S3, part B, shift represented by differences in the position of the centre of the area calculated for the coordinates of the principles). Also, there was less dispersion of the coordinates for the nine principles for the female participants.

#### Stratification by professional experience

Differentiation by years in the profession (up to or more than 20 years of experience) showed no significant changes in the rankings compared to the overall group, apart from a switch in the order of rankings for “legal conformity” and “content validity.” Overall, there were clear and significant differences in the coordinates of the various principles (Supplementary Table S3, part C). For participants with fewer years of experience, “technical adequacy” was closer to the “one-dimensional” quadrant. In contrast, more extended work experience led to a more conservative opinion, with a smaller contribution of the Better_I_ values. More specifically, the possible observance of the respective principles may have exerted less influence on the assessments made by these participants (see Supplementary Table S3, part C and Fig. 3B). This was also true for the other quality principles.

#### Stratification by private and professional use of health apps

There was an overlap of 72% (275/382 participants) among those who stated to be using health apps in private or work-related settings (22%, 84/382) or denied their use in both settings (50%, 191/382). An additional 13.6% (52/382) admitted to using such apps only for work purposes but not in private or vice versa (14.4%, 55/382).

In principle, all quality aspects were again assigned to the “must-be” category regardless of whether apps were used in private or work-related contexts or not (Fig. 3C and D). The exception was again “resource efficiency” (indifferent quadrant) for both settings. Participants without professional or private app use saw the latter somewhat closer to the “must-be” quadrant.

Independent of app use in either private or work-related settings, “legal conformity” always occupied first place, and the four quality principles “technical adequacy,” “usability,” “resource efficiency,” and transparency were consistently ranked last (i.e., ranks 6 to 9, see Table 2 as well as Supplementary Table S3, parts D and E). This conformed to the overall group (Table 2 and Supplementary Table S3, part A).

For participants without work-related use (Table 2 and Supplementary Table S3, part E), the ranking of the other principles was also identical to the overall group, while for those not using health apps in private contexts, only the order of “practicality” and “risk adequacy” differed (ranks 4 and 3 overall vs 3 and 4 without private use). For participants with private app use, there was a deviation from the participants overall by one position for “practicality” and “risk adequacy” and by two places for “ethical soundness.” For those with work-related app use, “practicality,” “risk adequacy,” and additionally “content validity” were downgraded by one rank compared to the unstratified group of participants. At the same time, “ethical soundness” was upvalued by three places.

On average, participants with both private and professional use of health apps had higher Better_I_ scores than those without (see centre coordinates shown in Fig. 3C and D).

For the Worse_I_ values, the analysis must be more differentiated: Here, without professional use, the values for “legal conformity” (at − 0.82 vs − 0.76 significantly), “resource efficiency” (− 0.41 vs − 0.38), and “transparency” (− 0.60 vs − 0.59) were more pronounced on the Worse_I_ axis than with such use and were thus more strongly demanded (Supplementary Table S3, part E). For those not using apps in private settings, this was again true for the same three principles of “legal conformity” (− 0.80 vs − 0.78), “resource efficiency” (− 0.42 vs 0.35), as well as transparency (− 0.61 vs − 0.58).

#### Stratification by interest in digitisation

For assessing the quality principles with respect to the participants’ stated interest in digitisation, the original 5-point Likert scaled answer options (“highly interested,” *n* = 157; “interested,” *n* = 159; “neutral,” *n* = 44; “less interested,” *n* = 16; “no interest,” *n* = 6) were summarised as “(highly) interested,” “neutral,” and “little to no interest” (Table 1 and Fig. 3E).

We did this for two reasons: on the one hand, to allow for easier comparison between the groups and, on the other hand, because the group sizes of those with little or no stated interest in digitisation would otherwise have been too small to even be suggestive of a tendency within these groups. At the other end of the scale, when looking at the “highly interested” and “interested participants” separately, there were only minor differences in the distribution of the satisfaction and dissatisfaction coefficients between both groups. Given these two factors, we decided to aggregate the abovementioned interest-related strata.

Stated interest in digital topics seemed to only slightly influence the ranking, although this similarity may be explained by the group’s relatively large number of participants. Among interested participants, the ranks of “practicality,” “risk adequacy,” and “ethical soundness” differed only slightly from the overall group (ranks 5, 4, and 3 vs 4, 3, and 5, see Table 2 and Supplementary Table S3, part F).

For disinterested participants, there were deviations in rank for “legal conformity” and “content validity” (rank 1 and 2, respectively), and “technical adequacy” and “usability” (ranks 6 and 8, respectively). “Legal conformity,” “content validity,” and “risk adequacy” ranked highest among participants with and without interest. Ranks for those who had a neutral attitude towards digitisation differed in that for these participants, “practicality” ranked first, however, again, followed by “legal conformity” and “content validity.” In all three strata, “usability,” “transparency,” “technical adequacy,” and “resource efficiency” again ranked last (albeit in a different order). A notable difference was that, in the case of disinterested participants, contrary to the assessment of the other strata and the overall group, the principles were almost exclusively located in the “indifferent” quadrant or, at best, on the borderline between “indifferent” and “must-be” (especially: “legal conformity,” “content validity,” “risk adequacy,” Fig. 3E).

## Discussion

### Principal findings

These category assignments and the perceived relevance values obtained in this study correspond to the results of our previous work. Then, as now, the participants of the respective evaluations assessed the quality principles to be (highly) relevant. In the field of internal medicine [11], the participants saw the quality principles as “very important” or “important” in 83 to 98% of ratings, with only resource efficiency given considerably fewer such ratings (61.6%). In comparison, for our present data, the quality principles were seen as being (very) important by between 84 to 97% of the participants, again with resource efficiency standing out (68.1% rated this as “important” or “very important”).

Using the “in-line-of-sight” method [13], the nine quality principles could, however, be consistently differentiated and ranked. While “legal conformity,” “content validity,” and “risk adequacy” were prioritised highest, “technical adequacy,” “usability,” as well as “resource efficiency,” and “transparency” were consistently ranked last.

Descriptively, the assignments were largely stable, apparently irrespective of demographic factors. Only a lack of interest in digitisation topics seemed to have a meaningful influence. The corresponding participants rated all quality principles indifferently, albeit this may not be fully representative due to the small number of participants. Nevertheless, an explanation for this notable deviation could be that a lack of interest meant that the participants were unable to transfer their quality expectations or their awareness of the need to observe basic quality principles in health care, which they almost certainly live by in everyday medical practice, to the digital domain. On the other hand, while one might suspect that the low response rate of our survey (4%) may represent a general lack of interest in the topic, other factors may be at play here. In addition to time constraints, a lack of opportunity or the necessary skill set for answering a digital survey, it is conceivable that some of those who did not respond refrained from participating out of excessive caution in the digital world, despite actually being interested. Nevertheless, had we obtained data for such individuals by other means, this might have influenced the rankings for the interested group. However, we suspect that only little would have changed with respect to the quality principles still being assigned to the must-be quadrant.

Apart from the notable differences concerning interest in digitisation, other demographics only seemed to have a negligible influence. This may, however, in part also have been due to the large proportion of those with an indicated interest in digitisation topics, possibly outweighing the other influences, as the majority of participants were either interested in the matter of digitisation (82.7%, 316/382) or had at least a neutral opinion (11.5%, 44/382) in this regard, with similar prioritizations of the nine quality principles. These participants apparently recognised the principles of “legal conformity,” “content validity,” and “risk adequacy” as essential to their daily practice. They were presumably able to transfer these to the discussion of digitisation topics. The current debate on data protection (in the context of “legal conformity”) with supposedly or actually reported health or content-related, as well as technical and legal risks, may support this [38–40]. Despite the strong influence of interest in digital topics, there are still a few points worth mentioning for the other factors. For example, based on the average values for the Better_I_ and Worse_I_ ratings, among those who reported private or professional use of health-related apps (Supplementary Table 3, parts D and E) and Fig. 3C and D), the presence (represented by the Better_I_ values) exerted more influence on the assessments than the absence of the principles (Worse_I_ values). Also, without private use of health-related apps, any lack of “legal conformity,” “resource efficiency,” and “transparency” was more negatively connoted than among those who used such apps in a private context.

Similarly, for professional use, this was true for “legal conformity” and “transparency.” It may be precisely these aspects that have so far prevented the participants in question from using apps in either setting. Information on “legal conformity” or information that supports the idea of transparency is usually found even less frequently in the provided app store information [11] than information for the other quality principles.

On the surface, the high rate (82.7%) of those who expressed an interest in digitisation might be perceived as being in contradiction to the low rate of those actually using health-related apps for personal (36.4%) or work-related (35.6%) matters. We do, however, not believe that to be the case. Those who enthusiastically use their smart devices for various (non-health) work-related or private purposes may still refrain from using health apps unless they absolutely have to, e.g., for health reasons of their own. Data published by Deloitte for clinicians using different types of digital technologies in care delivery [41] show the relatively low health app usage rate is similar for German physicians, especially compared to their European peers. Apps specifically targeting clinicians (possibly surpassing our participant’s perception of health apps due to an administrative or other focus) were used by 44% of German participants. The rate for patient apps or wearables was even lower at 21% [41].

Finally, the almost universal last-place ranking of “resource efficiency” seems understandable against the backdrop of increasingly powerful mobile devices. Apps commonly used in healthcare are unlikely to overtax today’s powerful mobile devices.

## Limitations

The return rate was low at approximately 4%. It is uncertain whether the respondents were representative of all physicians active in orthopaedics and trauma surgery in Germany, although there were only a few participants from other countries (10/382, 2.6%, the question was not answered by 2 participants). There was, however, only limited information about the demographics of the overall group of physicians active in this field: Numbers provided by the German medical association for the end of 2020 [42] showed an underrepresentation of female just physicians in the field (17.7% or 3077 out of 17,372 physicians officially active in this field, or, including those no longer active due to retirement or other reasons, 17.6% or 3611 out of 20,477). This was almost mirrored in our study, where, with 14% of female participants, we came relatively close to the overall ratio of female to male physicians in the field described in [42]. Unfortunately, we did not have access to the demographic data of the members of the two German professional orthopaedics associations to assess representability further. For other aspects, e.g. the number of retirees (19/382, 5%), our ratios were not as close to the overall numbers (17.6% or 3611 out of 20,477) of the orthopedists and trauma surgeons listed as retired or inactive due to other reasons in [42]. Compared to German orthopaedists and trauma surgeons overall, our ratios for those working in acute care and rehabilitation centre settings were also somewhat skewed vs those working in ambulatory settings (see the numbers for “work setting” in Table 1 for our participants; Germany overall: ambulatory setting 7675/17372 or 44.2%, inpatient settings 8869/17372 or 51.1%, other settings 4.8%, see [42]). We could not identify official data for factors such as age and professional experience for comparison.

Our findings may be somewhat distorted for the smaller strata, e.g. those with little interest in the topic (22/382 or 5.8%). Several factors may contribute to this. Participants lacking interest in digitisation may have felt uncomfortable being approached by email, and there may also have been those who generally struggle with or like to avoid online questionnaires. Additionally, there may be a bias to the detriment of younger participants, presumably more experienced in the app context as “digital natives.”

It was also impossible to rule out concurrent membership in both associations, possibly resulting in multiple invitations for participation.

Future work will need to deal with an evaluation on a larger scale and for additional areas of application, e.g., with adaptations to the rather generic list of quality principles used as a basis for the work presented here. In addition, an investigation of the comparability of explicitly asked rankings with the implicit orders of the examined items determined in the manner described here is pending. Furthermore, the recruitment strategy could, for example, be improved by offering incentives to increase the sample size. These could include material benefits such as compensation for the invested time spent on the survey.

## Conclusions

The presented ranking method allows the differentiation of equally categorised elements and permits prioritisation. In practical terms, 8 out of 9 quality principles are considered prerequisites for quality. Still, among them, “legal conformity,” “content validity,” and “risk adequacy” rank highest, with the smallest variation across demographics. Compared to previous studies conducted in other medical fields [11, 12], the relevance ratings are essentially similar. We believe this can be seen as evidence of a fundamental understanding of quality aspects in the medical environment.

There are indications that interest in digitisation topics (or a lack thereof) may influence the results. This could be a pivotal point for professional associations (and, in turn, their members) to advance the idea of digitisation in the medical field. By highlighting the potential benefits of digitisation and supporting their members in strengthening their digital skills, e.g., by providing appropriate training materials and tools related to quality in the digital domain, they can improve their position vis-à-vis other players in the field of medicine. One factor that might fuel this could be that those better educated in this regard might also be more driven and encouraged to participate in the relevant discussions both within and outside their professional societies.

## Supplementary Information

**Additional file 1: Table S1.** Quality principles and corresponding functional and dysfunctional questions as required by the Kano model (translated from the original German-language version). The table has been copied from [1].

**Additional file 2: Table S2.** Questions regarding the relevance of each of the nine quality principles (translated from the original German version). The table has been copied from [1].

**Additional file 3: Table S3.** Parameters and rankings for the nine quality principles (based on the distance to must-be-corner and angle to the right outer boundary) using the ‘in-line-of-sight’ method. A all *N* = 382 participants (unstratified). Stratifications are shown in parts B (gender), C (work experience), D (use of health apps in private settings), E (use of health apps for work-related purposes), and F (interest in digitisation).

## Data Availability

The datasets generated and/or evaluated during the current study are not publicly available because they were only collected in the German language and are difficult to analyse and process by non-German-speaking interested parties without further explanation. They are, however, available from the corresponding author on reasonable request.
