# Supplementary material for: Orthopaedic and trauma surgeons’ prioritisation of app quality principles based on their demographic background
Source: BMC Musculoskelet Disord. 2023 Feb 23;24:146. doi: 10.1186/s12891-023-06226-y (PMC9948494; doi:10.1186/s12891-023-06226-y)
Supplement: Supplementary file 1 — Additional file 1: Table S1. Quality principles and corresponding functional and dysfunctional questions as required by the Kano model (translated from the original German-language version). The table has been copied from [1]. [file 12891_2023_6226_MOESM1_ESM.docx]

# Supplementary Table 1

Table S1. Quality principles and corresponding functional and dysfunctional questions as required by the Kano model (translated from the original German-language version). The table has been copied from (1).

| Principle | Functional question | Dysfunctional question |
| --- | --- | --- |
| Practicality | What would you say if apps could be used for the intended purpose? | What would you say if apps could not be used for the intended purpose? |
| Risk adequacy | What would you say if apps did not pose a disproportionate health, social, or economic risk to users? | What would you say if apps posed disproportionate health, social, or economic risks to users? |
| Ethical soundness | What would you say if discrimination and stigmatization were avoided when developing, offering, and using apps? | What would you say if discrimination or stigmatization were not avoided when developing, offering, operating, and using apps? |
| Legal conformity | What would you say if apps were compliant with data protection regulations as well as professional and health regulations? | What would you say if apps failed to comply with data protection, professional, or health regulations? |
| Content validity | What would you say if the content used in apps was valid and trustworthy? | What would you say if the content used in apps was not valid or not trustworthy? |
| Technical adequacy | What would you say if apps were easy to maintain and could be used independent of a specific platform? | What would you say if apps were hard to maintain or could not be used independent of a specific platform? |
| Usability | What would you say if apps were designed and implemented according to the requirements of the target group(s)? | What would you say if apps were not designed and implemented to meet the needs of the target group(s)? |
| Resource efficiency | What would you say if apps were to use resources such as battery and computing power efficiently? | What would you say if apps made only inefficient use of resources such as battery or computing power? |
| Transparency | What would you say if apps provided transparent information about inherent quality features? | What would you say if apps did not provide transparent information about inherent quality characteristics? |

# References

1. Malinka C, von Jan U, Albrecht U-V. Prioritization of quality principles for health apps using the KANO model: survey study. JMIR Mhealth Uhealth 2022 Jan;10(1):e26563. Available from <https://mhealth.jmir.org/2022/1/e26563>. Applicable License: Creative Commons Attribution License (https://creativecommons.org/licenses/by/4.0/), which permits unrestricted use, distribution, and reproduction in any medium, provided the original work, first published in JMIR mHealth and uHealth, is properly cited.
