# Supplementary material for: Orthopaedic and trauma surgeons’ prioritisation of app quality principles based on their demographic background
Source: BMC Musculoskelet Disord. 2023 Feb 23;24:146. doi: 10.1186/s12891-023-06226-y (PMC9948494; doi:10.1186/s12891-023-06226-y)
Supplement: Supplementary file 2 — Additional file 2: Table S2. Questions regarding the relevance of each of the nine quality principles (translated from the original German version). The table has been copied from [1]. [file 12891_2023_6226_MOESM2_ESM.docx]

# Supplementary Table 2

Table S2. Questions regarding the relevance of each of the nine quality principles (translated from the original German version). The table has been copied from (1).

| Principle | Perceived relevance |
| --- | --- |
| Practicality | How important is it to you that apps can be used for the intended purpose? |
| Risk adequacy | How important is it to you that apps are low risk in terms of health, social, or economic risks? |
| Ethical soundness | How important is it to you to avoid discrimination and stigmatization when developing, offering, operating, and using apps? |
| Legal conformity | How important is it to you that data protection, professional, and health regulations are respected in apps? |
| Content validity | How important is the validity and trustworthiness of the health-related content presented and used in an app to you? |
| Technical adequacy | How important are easy maintainability and platform-independent or cross-platform usability of apps to you? |
| Usability | How important is the target group–oriented design and operation of apps to you? |
| Resource efficiency | How important to you is the efficient use of resources through apps, for example in terms of battery and computing power? |
| Transparency | How important is it to you that apps provide transparent information about inherent quality features? |

# References

1. Malinka C, von Jan U, Albrecht U-V. Prioritization of quality principles for health apps using the KANO model: survey study. JMIR Mhealth Uhealth 2022 Jan;10(1):e26563. Available from <https://mhealth.jmir.org/2022/1/e26563>. Applicable License: Creative Commons Attribution License (https://creativecommons.org/licenses/by/4.0/), which permits unrestricted use, distribution, and reproduction in any medium, provided the original work, first published in JMIR mHealth and uHealth, is properly cited.
