# Supplementary material for: Orthopaedic and trauma surgeons’ prioritisation of app quality principles based on their demographic background
Source: BMC Musculoskelet Disord. 2023 Feb 23;24:146. doi: 10.1186/s12891-023-06226-y (PMC9948494; doi:10.1186/s12891-023-06226-y)
Supplement: Supplementary file 3 — Additional file 3: Table S3. Parameters and rankings for the nine quality principles (based on the distance to must-be-corner and angle to the right outer boundary) using the ‘in-line-of-sight’ method. A all N = 382 participants (unstratified). Stratifications are shown in parts B (gender), C (work experience), D (use of health apps in private settings), E (use of health apps for work-related purposes), and F (interest in digitisation). [file 12891_2023_6226_MOESM3_ESM.docx]

# Supplementary Table S3

Table S3. Parameters and rankings for the nine quality principles (based on the distance to must-be-corner and angle to the right outer boundary) using the ‘in-line-of-sight’ method. A all N=382 participants (unstratified). Stratifications are shown in parts B (gender), C (work experience), D (use of health apps in private settings), E (use of health apps for work-related purposes), and F (interest in digitisation).

| S3, part A: All N=382 participants (unstratified) | | | | | | | | | | | | | | | | | | | | | | | | | | | | | |
| --- | --- | --- | --- | --- | --- | --- | --- | --- | --- | --- | --- | --- | --- | --- | --- | --- | --- | --- | --- | --- | --- | --- | --- | --- | --- | --- | --- | --- | --- |
| Principle | | Worse_I_ | | Better_I_ | | Distance *d* | | Angle $\boldsymbol{\alpha}$ | | Factor *f* | | Rank | |  |  |  | |  | | |  | | |  | | |  | | |
| practicality | | -0.803 | | 0.246 | | 0.316 | | 51.3 | | 0.356 | | 4 | |  |  |  | |  | | |  | | |  | | |  | | |
| risk adequacy | | -0.834 | | 0.23 | | 0.284 | | 54.2 | | 0.326 | | 3 | |  |  |  | |  | | |  | | |  | | |  | | |
| ethical soundness | | -0.709 | | 0.197 | | 0.352 | | 34.2 | | 0.378 | | 5 | |  |  |  | |  | | |  | | |  | | |  | | |
| legal conformity | | -0.794 | | 0.126 | | 0.241 | | 31.4 | | 0.266 | | 1 | |  |  |  | |  | | |  | | |  | | |  | | |
| content validity | | -0.879 | | 0.211 | | 0.243 | | 60.1 | | 0.29 | | 2 | |  |  |  | |  | | |  | | |  | | |  | | |
| technical adequacy | | -0.656 | | 0.375 | | 0.509 | | 47.4 | | 0.546 | | 8 | |  |  |  | |  | | |  | | |  | | |  | | |
| usability | | -0.686 | | 0.302 | | 0.436 | | 43.8 | | 0.47 | | 6 | |  |  |  | |  | | |  | | |  | | |  | | |
| resource efficiency | | -0.397 | | 0.296 | | 0.672 | | 26.1 | | 0.693 | | 9 | |  |  |  | |  | | |  | | |  | | |  | | |
| transparency | | -0.599 | | 0.274 | | 0.486 | | 34.3 | | 0.513 | | 7 | |  |  |  | |  | | |  | | |  | | |  | | |
| S3, part B: Stratified by gender (adapted from (12)) | | | | | | | | | | | | | | | | | | | | | | | | | | | | | |
|  | | ***Females (A, n=54)*** | | | | | | | | | | | | ***Males (B, n=328)*** | | | | | | | | | | | | | ***A to B*** | | |
| Principle | | Worse_I_ | | Better_I_ | | Distance *d* | | Angle $\alpha$ | | Factor *f* | | Rank | | Worse_I_ | Better_I_ | Distance *d* | | Angle $\alpha$ | | | Factor *f* | | | Rank | | | Dist. | | |
| practicality | | -0.82 | | 0.32 | | 0.37 | | 60 | | 0.41 | | 5 | | -0.80 | 0.23 | 0.31 | | 50 | | | 0.35 | | | 4 | | | 0.0853* | | |
| risk adequacy | | -0.90 | | 0.26 | | 0.28 | | 69 | | 0.34 | | 3 | | -0.82 | 0.22 | 0.29 | | 52 | | | 0.33 | | | 3 | | | 0.0856* | | |
| ethical soundness | | -0.81 | | 0.25 | | 0.31 | | 52 | | 0.35 | | 4 | | -0.69 | 0.19 | 0.36 | | 32 | | | 0.39 | | | 5 | | | 0.1295* | | |
| legal conformity | | -0.87 | | 0.19 | | 0.23 | | 56 | | 0.28 | | 2 | | -0.78 | 0.12 | 0.25 | | 28 | | | 0.27 | | | 1 | | | 0.1153* | | |
| content validity | | -0.94 | | 0.18 | | 0.19 | | 70 | | 0.24 | | 1 | | -0.87 | 0.22 | 0.25 | | 59 | | | 0.30 | | | 2 | | | 0.0772* | | |
| technical adequacy | | -0.62 | | 0.42 | | 0.57 | | 48 | | 0.61 | | 8 | | -0.66 | 0.37 | 0.50 | | 47 | | | 0.54 | | | 8 | | | 0.0703 | | |
| usability | | -0.68 | | 0.35 | | 0.48 | | 47 | | 0.52 | | 6 | | -0.69 | 0.29 | 0.43 | | 43 | | | 0.46 | | | 6 | | | 0.0617 | | |
| resource efficiency | | -0.46 | | 0.42 | | 0.68 | | 38 | | 0.71 | | 9 | | -0.39 | 0.28 | 0.67 | | 24 | | | 0.69 | | | 9 | | | 0.1598* | | |
| transparency | | -0.60 | | 0.35 | | 0.53 | | 42 | | 0.57 | | 7 | | -0.60 | 0.26 | 0.48 | | 33 | | | 0.50 | | | 7 | | | 0.0944* | | |
| * Worse_I_ and Better_I_ coordinates differ significantly between the strata, i.e. Euclid. distance > 0.05 · √ 2 ≈ 0.0707 | | | | | | | | | | | | | | | | | | | | | | | | | | | | | |
| *Table S3 (continued)* | | | | | | | | | | | | | | | | | | | | | | | | | | | | | |
| S3, part C: Work experience | | | | | | | | | | | | | | | | | | | | | | | | | | | | | |
|  | | ***Up to 20 years (A, n=169)*** | | | | | | | | | | | | ***More than 20 years (B, n=213)*** | | | | | | | | | | | | | | | ***A to B*** |
| Principle | | Worse_I_ | | Better_I_ | | Distance *d* | | Angle $\alpha$ | | Factor *f* | | Rank | | Worse_I_ | Better_I_ | | Distance *d* | | | Angle $\alpha$ | | | Factor *f* | | | Rank | | | Dist. |
| Practicality | | -0.828 | | 0.301 | | 0.347 | | 60.2 | | 0.395 | | 4 | | -0.783 | 0.2041 | | 0.298 | | | 43.2 | | | 0.332 | | | 4 | | | 0.1071* |
| risk adequacy | | -0.854 | | 0.301 | | 0.335 | | 64.2 | | 0.385 | | 3 | | -0.818 | 0.1762 | | 0.253 | | | 44.1 | | | 0.288 | | | 3 | | | 0.1303* |
| ethical soundness | | -0.690 | | 0.262 | | 0.407 | | 40.2 | | 0.438 | | 5 | | -0.724 | 0.1472 | | 0.313 | | | 28.1 | | | 0.335 | | | 5 | | | 0.1203* |
| legal conformity | | -0.826 | | 0.167 | | 0.242 | | 43.8 | | 0.276 | | 1 | | -0.770 | 0.0942 | | 0.249 | | | 22.2 | | | 0.266 | | | 2 | | | 0.0922* |
| content validity | | -0.914 | | 0.267 | | 0.280 | | 72.2 | | 0.337 | | 2 | | -0.851 | 0.1677 | | 0.224 | | | 48.5 | | | 0.262 | | | 1 | | | 0.1174* |
| technical adequacy | | -0.638 | | 0.447 | | 0.575 | | 51.0 | | 0.615 | | 8 | | -0.669 | 0.3190 | | 0.460 | | | 43.9 | | | 0.494 | | | 8 | | | 0.1318* |
| usability | | -0.669 | | 0.368 | | 0.495 | | 48.0 | | 0.532 | | 6 | | -0.699 | 0.2501 | | 0.392 | | | 39.7 | | | 0.423 | | | 6 | | | 0.1213* |
| resource efficiency | | -0.383 | | 0.370 | | 0.719 | | 31.0 | | 0.743 | | 9 | | -0.406 | 0.2410 | | 0.641 | | | 22.1 | | | 0.658 | | | 9 | | | 0.1309* |
| transparency | | -0.557 | | 0.330 | | 0.552 | | 36.7 | | 0.581 | | 7 | | -0.630 | 0.2303 | | 0.435 | | | 31.9 | | | 0.461 | | | 7 | | | 0.124* |
| * Worse_I_ and Better_I_ coordinates differ significantly between the strata, i.e. Euclid. Distance > 0.05 · √ 2 ≈ 0.0707 | | | | | | | | | | | | | | | | | | | | | | | | | | | | | |
| S3, part D: Use of health-related apps in private settings | | | | | | | | | | | | | | | | | | | | | | | | | | | | | |
|  | ***Private use (A, n=139)*** | | | | | | | | | | | | ***No private use (A, n=243)*** | | | | | | | | | | | | | | | ***A to B*** | |
| Principle | Worse_I_ | | Better_I_ | | Distance *d* | | Angle $\alpha$ | | Factor *f* | | Rank | | Worse_I_ | | Better_I_ | Distance *d* | | | Angle $\alpha$ | | | Factor *f* | | | Rank | | | Dist. | |
| practicality | -0.808 | | 0.342 | | 0.392 | | 60.7 | | 0.440 | | 5 | | -0.799 | | 0.192 | 0.278 | | | 43.8 | | | 0.312 | | | 3 | | | 0.1497* | |
| risk adequacy | -0.877 | | 0.281 | | 0.307 | | 66.3 | | 0.359 | | 4 | | -0.810 | | 0.202 | 0.278 | | | 46.8 | | | 0.314 | | | 4 | | | 0.1031* | |
| ethical soundness | -0.766 | | 0.221 | | 0.322 | | 43.4 | | 0.357 | | 3 | | -0.678 | | 0.184 | 0.371 | | | 29.7 | | | 0.395 | | | 5 | | | 0.0957* | |
| legal conformity | -0.777 | | 0.152 | | 0.269 | | 34.3 | | 0.296 | | 1 | | -0.804 | | 0.111 | 0.225 | | | 29.4 | | | 0.248 | | | 1 | | | 0.0488 | |
| content validity | -0.903 | | 0.256 | | 0.274 | | 69.3 | | 0.328 | | 2 | | -0.865 | | 0.185 | 0.229 | | | 53.8 | | | 0.271 | | | 2 | | | 0.0813* | |
| technical adequacy | -0.661 | | 0.449 | | 0.562 | | 53.0 | | 0.604 | | 8 | | -0.652 | | 0.333 | 0.482 | | | 43.7 | | | 0.516 | | | 8 | | | 0.1165* | |
| usability | -0.726 | | 0.313 | | 0.416 | | 48.7 | | 0.454 | | 6 | | -0.663 | | 0.295 | 0.448 | | | 41.2 | | | 0.480 | | | 6 | | | 0.0648 | |
| resource efficiency | -0.353 | | 0.316 | | 0.721 | | 26.0 | | 0.741 | | 9 | | -0.422 | | 0.284 | 0.644 | | | 26.2 | | | 0.665 | | | 9 | | | 0.0764* | |
| transparency | -0.577 | | 0.292 | | 0.513 | | 34.6 | | 0.541 | | 7 | | -0.611 | | 0.263 | 0.470 | | | 34.1 | | | 0.496 | | | 7 | | | 0.044 | |
| * Worse_I_ and Better_I_ coordinates differ significantly between the strata, i.e. Euclid. distance > 0.05 · √ 2 ≈ 0.0707 | | | | | | | | | | | | | | | | | | | | | | | | | | | | | |

| *Table S3 (continued)* | | | | | | | | | | | | | | | | | | | | |
| --- | --- | --- | --- | --- | --- | --- | --- | --- | --- | --- | --- | --- | --- | --- | --- | --- | --- | --- | --- | --- |
| S3, part E: Use of health-related apps in work settings | | | | | | | | | | | | | | | | | | | | |
|  | ***Work-related use (A, n=136)*** | | | | | | ***No work-related use (B, n=246)*** | | | | | | | | | | | | ***A to B*** | |
| Principle | Worse_I_ | Better_I_ | Distance *d* | Angle $\alpha$ | Factor *f* | Rank | Worse_I_ | | Better_I_ | | Distance *d* | | Angle $\alpha$ | | Factor *f* | | Rank | | Dist. | |
| practicality | -0.854 | 0.308 | 0.340 | 64.7 | 0.391 | 5 | -0.774 | | 0.214 | | 0.311 | | 43.4 | | 0.345 | | 4 | | 0.1235* | |
| risk adequacy | -0.838 | 0.275 | 0.319 | 59.5 | 0.366 | 4 | -0.831 | | 0.207 | | 0.267 | | 50.8 | | 0.307 | | 3 | | 0.0681 | |
| ethical soundness | -0.753 | 0.216 | 0.328 | 41.1 | 0.361 | 2 | -0.686 | | 0.188 | | 0.366 | | 30.8 | | 0.390 | | 5 | | 0.0728* | |
| legal conformity | -0.757 | 0.149 | 0.285 | 31.5 | 0.310 | 1 | -0.815 | | 0.113 | | 0.217 | | 31.4 | | 0.241 | | 1 | | 0.0684 | |
| content validity | -0.882 | 0.285 | 0.308 | 67.5 | 0.361 | 3 | -0.877 | | 0.170 | | 0.210 | | 54.2 | | 0.252 | | 2 | | 0.1147* | |
| technical adequacy | -0.661 | 0.420 | 0.540 | 51.2 | 0.580 | 8 | -0.652 | | 0.350 | | 0.493 | | 45.2 | | 0.529 | | 8 | | 0.071* | |
| usability | -0.697 | 0.339 | 0.455 | 48.2 | 0.493 | 6 | -0.680 | | 0.281 | | 0.426 | | 41.3 | | 0.458 | | 6 | | 0.0605 | |
| resource efficiency | -0.376 | 0.348 | 0.715 | 29.2 | 0.738 | 9 | -0.408 | | 0.268 | | 0.650 | | 24.3 | | 0.669 | | 9 | | 0.0866* | |
| transparency | -0.589 | 0.316 | 0.518 | 37.5 | 0.548 | 7 | -0.604 | | 0.251 | | 0.469 | | 32.4 | | 0.494 | | 7 | | 0.0666 | |
| * Worse_I_ and Better_I_ coordinates differ significantly between the strata, i.e. Euclid. distance > 0.05 · √ 2 ≈ 0.0707 | | | | | | | | | | | | | | | | | | | | |
| S3, part F: Interest in digitisation | | | | | | | | | | | | | | | | | | | | |
|  | ***(Highly) interested (A, n=316)*** | | | | | | | ***Neutral (B, n=44)*** | | | | | | | | | | | |  |
| Principle | Worse_I_ | Better_I_ | Distance *d* | Angle $\alpha$ | Factor *f* | Rank | | Worse_I_ | | Better_I_ | | Distance *d* | | Angle $\alpha$ | | Factor *f* | | Rank | |  |
| practicality | -0.83 | 0.28 | 0.33 | 58.35 | 0.38 | 5 | | -0.85 | | 0.1 | | 0.18 | | 33.2 | | 0.21 | | 1 | |  |
| risk adequacy | -0.87 | 0.26 | 0.29 | 62.73 | 0.34 | 3 | | -0.8 | | 0.16 | | 0.26 | | 37.87 | | 0.29 | | 5 | |  |
| ethical soundness | -0.72 | 0.21 | 0.35 | 38 | 0.38 | 4 | | -0.78 | | 0.14 | | 0.26 | | 32.97 | | 0.28 | | 4 | |  |
| legal conformity | -0.82 | 0.14 | 0.23 | 36.77 | 0.26 | 1 | | -0.82 | | 0.12 | | 0.22 | | 32.87 | | 0.25 | | 2 | |  |
| content validity | -0.91 | 0.23 | 0.25 | 68.28 | 0.3 | 2 | | -0.88 | | 0.17 | | 0.2 | | 55.45 | | 0.25 | | 3 | |  |
| technical adequacy | -0.67 | 0.4 | 0.51 | 50.67 | 0.55 | 8 | | -0.66 | | 0.33 | | 0.47 | | 44.76 | | 0.51 | | 7 | |  |
| usability | -0.72 | 0.32 | 0.43 | 49.05 | 0.47 | 6 | | -0.71 | | 0.26 | | 0.39 | | 42.26 | | 0.42 | | 6 | |  |
| resource efficiency | -0.41 | 0.3 | 0.67 | 26.93 | 0.69 | 9 | | -0.41 | | 0.27 | | 0.65 | | 24.4 | | 0.67 | | 9 | |  |
| transparency | -0.62 | 0.29 | 0.48 | 36.98 | 0.51 | 7 | | -0.61 | | 0.29 | | 0.49 | | 35.95 | | 0.51 | | 8 | |  |
|  | ***Little to no interest (C, n=22)*** | | | | | | |  | | | | | | | | | | | |  |
| Principle | Worse_I_ | Better_I_ | Distance *d* | Angle $\alpha$ | Factor *f* | Rank | |  | |  | |  | |  | |  | |  | |  |
| practicality | -0.43 | 0.06 | 0.57 | 5.85 | 0.57 | 4 | |  | |  | |  | |  | |  | |  | |  |
| risk adequacy | -0.48 | 0.06 | 0.52 | 6.56 | 0.53 | 3 | |  | |  | |  | |  | |  | |  | |  |
| ethical soundness | -0.39 | 0.08 | 0.62 | 7.82 | 0.62 | 5 | |  | |  | |  | |  | |  | |  | |  |
| legal conformity | -0.48 | 0 | 0.52 | 0 | 0.52 | 2 | |  | |  | |  | |  | |  | |  | |  |
| content validity | -0.51 | 0.03 | 0.49 | 3.76 | 0.49 | 1 | |  | |  | |  | |  | |  | |  | |  |
| technical adequacy | -0.4 | 0.17 | 0.62 | 16.26 | 0.63 | 6 | |  | |  | |  | |  | |  | |  | |  |
| usability | -0.25 | 0.13 | 0.76 | 9.62 | 0.77 | 8 | |  | |  | |  | |  | |  | |  | |  |
| resource efficiency | -0.24 | 0.27 | 0.81 | 19.37 | 0.82 | 9 | |  | |  | |  | |  | |  | |  | |  |
| transparency | -0.36 | 0.06 | 0.64 | 5.39 | 0.65 | 7 | |  | |  | |  | |  | |  | |  | |  |
